# Cervical screening attendance in young women and people with a cervix: An application of the COM‐B model

**DOI:** 10.1111/bjhp.70016

**Published:** 2025-08-18

**Authors:** Sonia Shpendi, Paul Norman, Jilly Gibson‐Miller, Rebecca Webster

**Affiliations:** ^1^ Department of Psychology University of Sheffield Sheffield UK; ^2^ Department of Education University of Sheffield Sheffield UK

**Keywords:** cervical cancer, cervical screening, COM‐B model, HPV vaccination, young women

## Abstract

**Objectives:**

Cervical cancer (CC), which is caused by the human papillomavirus (HPV), results in around 3000 new cancer cases yearly in the UK. According to recent figures, rates in the UK have increased by 13% in young women over the last decade; screening attendance has fallen to a 10‐year low. As the majority of women now reaching the screening age (24.5 years old) will be HPV vaccinated, research is needed to assess the possible impact of this successful immunisation programme on screening behaviours as well as to further our understanding of the current barriers and facilitators to screening and how these may differ between attendees and non‐attendees.

**Design:**

Cross‐sectional survey.

**Methods:**

Participants comprised 200 young women (attendees *n* = 100, non‐attendees *n* = 100) aged 25–30 years old living in the UK. Participants completed an online questionnaire assessing COM‐B model components and HPV vaccination status.

**Results:**

A multiple logistic regression analysis revealed that reflective motivation was the only COM‐B component that was a significant independent predictor of screening attendance, such that higher reflective motivation scores increased the odds of having attended cervical screening. In addition, HPV‐vaccinated individuals had significantly greater odds of having attended screening when compared to non‐vaccinated individuals.

**Conclusions:**

Reflective motivational factors are crucial in encouraging young women to attend CC screening. Future work should focus on developing interventions that enhance reflective motivation.

**Statement of Contribution**

What is already known about this subject?

- Previous research has examined several factors associated with CC screening uptake in young women, including knowledge and awareness, socioeconomic status, and healthcare access; as well as the positive impact of being HPV vaccinated on screening attendance.

What does this study add?

- Analysis of the COM‐B model components associated with cervical screening attendance.
- Analysis of the impact of the HPV vaccination on cervical screening attendance.

## INTRODUCTION

Globally, cervical cancer (CC) remains the fourth most common cancer in females, with 350,000 deaths worldwide each year (WHO, 2024). There are around 3000 new cases of CC a year in the UK. Epidemiological studies have revealed that the highest prevalence of preinvasive CC lesions, which without treatment may develop into invasive carcinoma, is in females aged 25–29 (Cancer Research UK, 2019). In addition, the peak incidence rate of CC has been observed in younger age groups, with 30–34‐year‐olds making up 15.2% of cases, closely followed by 25–29‐year‐olds making up 13.4% of cases (NHS England, 2023).

CC cases have declined over the last decade, partly due to the availability of the National Health Service (NHS) CC screening programme and the success of the HPV immunisation programme (Falcaro et al., 2021). In 2018, the World Health Organization called for coordinated global action to eliminate CC, ensuring that all girls are vaccinated against HPV and that at least 70% of women are screened by 35 years old (WHO, 2024). However, both attendance and vaccination rates have fallen in recent years (UK Health Security Agency, 2022; Urwin et al., 2024). For example, vaccination coverage in 2022 to 2023 was 4.4% lower compared to coverage in 2021 to 2022 (67.3% coverage rate), as well as 21% lower than pre‐pandemic levels (2018–2019) in England (UK Health Security Agency, 2024). Given that the HPV vaccine is only 90% effective, those vaccinated still require CC screening to mitigate this risk further (Falcaro et al., 2021). Moreover, the sizeable minority of young women who are unvaccinated may be at heightened risk, further reinforcing the need for CC screening.

Cervical screening programme uptake remains central to early diagnosis, as CC is asymptomatic and can go undetected for many years; 99.8% of CC cases are preventable with early detection (Roope, 2021). Currently, the NHS aims to achieve CC screening coverage of 80% of eligible individuals. In 2024, only 68.8% of those eligible attended screening within the last 3.5 years, which saw only a .1% increase from the previous year (NHS England, 2024). Moreover, younger age groups are far less likely to attend screening than older age groups (59.7% of 25–29‐year‐olds vs. 72.1% of 45–49‐year‐olds) (Digital, 2021). Therefore, many young women in the UK do not respond to their first screening invitation at the age of 25. First‐time screening experiences are particularly important as they can have a significant positive impact on the trajectory of future screening attendance. Evidence has shown that past behaviour is one of the strongest predictors of future behaviour (McEachan et al., 2011), highlighting the need to understand and address the barriers to initial screening attendance.

Previous research has examined several factors associated with CC screening uptake in young women, including knowledge and awareness (Ackerson et al., 2008; Enyan et al., 2022), socioeconomic status and healthcare access (Urwin et al., 2024). Similarly, a recent systematic review (Shpendi et al., 2025) highlighted several studies reporting a significant positive relationship between knowledge and screening attendance. In addition, those in a relationship, sexually active and/or HPV vaccinated were also more likely to have attended CC screening. The review also highlighted communication with friends as a prominent facilitator, and financial constraints, embarrassment, and low accessibility as key barriers to screening uptake.

Theoretical frameworks, like the COM‐B model of behaviour change, can provide an overarching framework to capture the factors that may influence CC screening behaviour and provide a structure for developing interventions to increase the uptake of CC screening (Michie et al., 2011). The COM‐B model states that for the behaviour to take place, an individual must have: (1) the physical and psychological capability to perform the behaviour; (2) the physical and social opportunity to do so; and (3) reflective (conscious thought and decision‐making) and automatic (habits and subconscious processes) motivation (Michie et al., 2011). The COM‐B model has been used to explain a range of health behaviours, including Covid‐19 protection behaviours (Gibson Miller et al., 2020), physical activity (Howlett et al., 2019) and healthy eating (Isbanner et al., 2024).

In relation to CC screening specifically, a previous qualitative study used the COM‐B model to interpret current barriers and facilitators for both attendees and non‐attendees in young women (Shpendi et al., 2024). Reflective motivations (e.g., reassurance) and automatic motivations (e.g., embarrassment) were key facilitators for attending CC screening in both groups. However, social opportunity factors (e.g., open communication) were reported predominantly among attendees, highlighting the need for more open communication amongst social circles for those who had not attended. Non‐attendees also reported automatic reflective motivations in the form of negative perceptions and past interactions with healthcare providers, contributing to their reluctance to participate in screenings. These barriers can also be heightened for those with comorbidities (Kiefe et al., 1998) or other health problems (Akinlotan et al., 2017). Similar to the previous review (Shpendi et al., 2025), participants did not report physical capability factors (e.g., not having the strength to attend screening) as barriers to attendance and therefore were not assessed in the current study.

The current study aims to compare young women and people with a cervix (aged 25–30 years old) who have received a CC screening invitation, examining the COM‐B components identified in a recent qualitative study as predictors of screening attendance (Shpendi et al., 2024). This age group was chosen to include those who are eligible for their first CC screening under the NHS cervical screening programme, whilst allowing for any delay in attendance. In addition, the study will also consider HPV vaccination status and demographic characteristics as potential predictors of CC screening attendance. The focus on invited individuals is crucial for understanding the factors influencing uptake within the eligible population. However, as it is possible for women of eligible age to attend screening without an invitation, and the fact that some women may have forgotten or mistakenly not received it, we also allowed the inclusion of participants who had not received a CC screening invitation but were eligible to attend screening and carried out a separate sensitivity analysis with these participants to see if this changed any of the findings.

## MATERIALS AND METHODS

### Sample recruitment

Participants were individuals based in the UK aged 25–30. Exclusion criteria included: individuals who have had a CC screening before being invited as part of the government cervical screening programme (i.e., before the age of 24), individuals with a personal history of CC or hysterectomy procedure, and individuals who cannot read or write in English.

Participants were recruited online using Prolific, using filters to specify the target population. These included age (25–30), sex (female) and area of residence (UK) to meet the eligibility criteria. In total, 5352 participants on Prolific were eligible for recruitment. Participants were recruited in two stages: first, a screening survey to assess inclusion/exclusion criteria and attendance status; and second, the main survey. Data were collected online through surveys hosted via Qualtrics. For the screening survey, 1000 participants were recruited. Eligible participants who met the inclusion/exclusion criteria were then split into attendees and non‐attendees, depending on screening questionnaire responses. This also included participants who were of the eligible age for screening but who had reported not yet receiving a cervical screening invitation. From these participants, all eligible attendees and non‐attendees were invited to participate in the main study until 100 in each group had been recruited. Data collection took place between 8 and 10 December 2023.

### Sample size

Based on a previous study that examined psychological factors associated with screening attendance (e.g., perceived efficacy and awareness of CC screening), an odds ratio of 2.00 was used as an estimate of the likely effect size for differences between attendees and non‐attendees (Hansen et al., 2011). A power calculation indicated that the minimum sample size to detect a small to medium effect size/odds ratio of 2.00 with 90% power is 148 participants. To account for dropouts and poor‐quality data, this was increased to 200 (100 attendees and 100 non‐attendees).

### Measures

A screening questionnaire was used to identify eligible participants based on the inclusion/exclusion criteria (i.e., “Have you ever been diagnosed with cervical cancer, had a hysterectomy procedure and/or had a positive pap smear result (if applicable)?” and “Did you attend your cervical screening before the age of 24.5 years old?”) and to obtain responses regarding attendance status (attendee or non‐attendee) (i.e., “Have you attended a cervical cancer screening? (also known as a pap smear)?”) and invitation status (“Have you received your cervical screening invitation letter?”).

The main questionnaire included questions regarding education (“What is the highest degree or level of education you have completed?”), relationship status (“What is your current relationship status?”), religious status (“What religion do you identify most with?”), HPV vaccination status (“Have you had the HPV vaccine?”), self‐rated health (“How would you rate your health status?”) (Bowling, 2005) and identification of any long‐standing illness, disability, or infirmity (Manor et al., 2001). Prolific provided further demographic data (i.e., employment status and age).

The COM‐B model components were assessed using a structure by Keyworth et al. (2020) and including factors identified in a previous systematic review (Shpendi et al., 2025) and qualitative study (Shpendi et al., 2024). Items were rated on an 11‐point scale ranging from “−5” (strongly disagree) to “+5” (strongly agree) and averaged to produce a scale score for each COM‐B model component (see Supporting Information for the full list of COM‐B model items included). The first item of each COM‐B component was also accompanied by a brief description, as recommended by Keyworth et al. (2020) (see Supporting Information for full questionnaire). Physical capability was not assessed in the current study, as it was not highlighted as an important component in an earlier qualitative study with the same target population (Shpendi et al., 2024).

*Psychological capability* was assessed using a general statement adapted from Keyworth et al. (2020) (i.e., “I am psychologically able to attend cervical cancer screening”) followed by nine items on different aspects of psychological capability to attend (e.g., “I know what cervical cancer screening is for”). Cronbach's alpha for the scale was .74.

*Social opportunity* was assessed using a general statement (i.e., “I have the social opportunity to attend a cervical cancer screening”) followed by six items related to social opportunity (e.g., “I am comfortable discussing cervical cancer screening experiences with my social circle”). Cronbach's alpha for the scale was .76.

*Physical opportunity* was assessed using a generic statement (i.e. “I have the physical opportunity to attend cervical cancer screening”) followed by four items related to physical opportunity (e.g., “Booking a cervical cancer screening is difficult for me”). Cronbach's alpha for the scale was .72.

*Reflective motivation* was assessed using a generic statement (i.e., “I am motivated to attend cervical cancer screening”) followed by nine items reflecting reflective motivation (e.g., “Cervical cancer screening is important”). Cronbach's alpha for the scale was .85.

*Automatic motivation* was assessed using a generic statement (i.e., “Attending cervical cancer screening is something that I do automatically”) followed by five items assessing aspects of automatic motivation (e.g., “Cervical cancer screening is embarrassing for me”). One item (“I am scared of the cervical cancer screening procedure”) was removed to improve reliability from .61 to .70.

### Analysis

SPSS (Version 29) was used for statistical analysis. Descriptive analyses were used to describe sample characteristics. The two groups (attendees vs. non‐attendees) were compared on their demographic characteristics and the COM‐B model components using unadjusted (i.e., univariate) logistic regression analysis. Demographic variables that reached a *p*‐value of < .05 were then subsequently controlled for in a multiple logistic regression containing all COM‐B model components. The degree of association between independent variables (e.g., COM‐B model components) and the dependent variable (i.e., attendance versus non‐attendance) was analysed using odds ratios with 95% confidence intervals. Statistical significance was observed at *p* < .05. Additional analyses reported in the Supporting Information also examined associations between each of the COM‐B model items and screening attendance. The main analysis included only those who had received a cervical screening invitation letter (*N* = 179, *n* = 96 attendees & *n* = 73 non‐attendees). However, as individuals who have not received a cervical screening invitation may face different barriers and experiences to attending CC screening, we included participants who have not received a cervical screening invitation as part of a sensitivity analysis to see if this altered any of the findings with CC attendance (*N* = 200, *n* = 100 attendees & *n* = 100 non‐attendees) (see Supporting Information).

## RESULTS

### Participant characteristics

In total, 179 participants aged 25–30 were recruited for the main study, comprising 96 attendees and 83 non‐attendees. The mean age of participants was 27.65 (SD = 1.76). The majority of participants identified as female (96%), were white (79.9%) and had attended higher education (education beyond school level) (78.8%). Most participants reported that they were HPV vaccinated (70.9%), with 54% reporting having received two or more doses. Demographic details are provided in Table 1. For full demographic frequencies including those who had not received a cervical screening invitation, see Supporting Information.

**TABLE 1:** Demographic data and associations with screening attendance.

Variable	Attendees	Non‐ attendees	Total	Unadjusted OR (95% CI)	*p*
	*n*	*n*	*n* (%)		
Gender					
Female	94	78	172 (96.1)	3.01 (.57–15.96)	.195
Prefer self‐identify	2	5	7 (3.9)		
Ethnicity					
White British or White Other	80	63	143 (79.9)	1.587 (.76–3.31)	.218
Other	16	20	36 (20.1)		
Place of residence					
England	88	72	160 (89.4)	1.68 (.64–4.40)	.290
Scotland, Northern Ireland and Wales	8	11	19 (10.6)		
Education					
Higher education	81	60	141 (78.8)	2.07 (1.00–4.30)	.051
School‐level education	15	23	38 (21.2)		
Relationship status					
Partnered	63	40	103 (57.5)	2.05 (1.12–3.75)	.019*
Other	33	43	76 (42.5)		
Religion					
Not religious	70	54	124 (69.3)	.71 (.37–1.38)	.312
Religious	24	26	50 (27.9)		
Prefer not to say^a^	2	3	5 (2.8)		
Employment status					
Employed	74	47	121 (67.6)	3.15 (1.31–3.15)	.011*
Not employed	9	18	27 (15.1)		
Missing^a^	13	18	31 (17.3)		
Long‐standing illness, disability or infirmity					
No	87	63	150 (83.8)	.33 (.14–.76)	.010*
Yes	9	20	29 (16.2)		
HPV vaccinated					
Yes	74	53	127 (70.9)	1.78 (.88–3.63)	.110
No	18	23	41 (22.9)		
Not sure	4	7	11 (6.1)		
Number of doses					
Two or more	59	38	97 (54.2)	2.07 (.44–9.77)	.358
One	3	4	7 (3.9)		
Not sure^a^	12	11	23 (12.8)		

	Mean (SD)	Mean (SD)	Mean (SD)	Unadjusted OR (95% CI)	*p*
Age	27.84 (1.76)	27.42 (1.73)	27.65 (1.76)	1.15 (.97–1.36)	.109
Health status	2.48 (.74)	2.58 (.80)	2.5 (.77)	.84 (.57–1.24)	.388

^a^ Don't know/not sure/missing responses were excluded from the analysis.

* *p* < .05.

### Demographic factors associated with screening attendance

The odds of having attended CC screening were significantly associated with being partnered (OR, 2.05; 95% CI, 1.12–3.75) and being in employment (OR, 3.15; 95% CI, 1.31–3.15). In contrast, having a long‐standing illness or disability (OR, .33; 95% CI, .14–.76) was negatively associated with having attended CC screening (see Table 1).

### COM‐B model components associated with screening attendance

Table 2 presents the results of the unadjusted (univariate) logistic regression analyses testing associations between each COM‐B model component and CC screening attendance. All of the COM‐B model components were found to have a significant association with CC screening attendance. Increased scores in Psychological Capability (OR, 1.93; 95% CI, 1.45–2.60), Social Opportunity (OR, 1.47; 95% CI, 1.24–1.74), Physical Opportunity (OR, 1.98; 95% CI, 1.53–2.56), Reflective Motivation (OR, 2.91; 95% CI, 2.17–3.90) and Automatic Motivation (OR 2.28; 95% CI, 1.76–2.96) significantly increased the odds of attending screening. Additional unadjusted (univariate) logistic regression analyses were conducted to test associations between each of the reflective motivation items and CC screening attendance. Seven (out of ten) of the items were predictive of CC screening attendance (see Supporting Information).

**TABLE 2:** COM‐B components and associations with screening attendance.

Variable	Attendees	Non‐attendees	Unadjusted OR (95% CI)	*p*‐value
	Mean (SD)	Mean (SD)		
Psychological capacity	3.35 (.97)	2.57 (1.24)	1.93 (1.42–2.60)	<.001*
Social opportunity	2.50 (1.73)	.99 (2.19)	1.47 (1.24–1.74)	<.001*
Physical opportunity	3.90 (1.14)	2.78 (1.36)	1.98 (1.53–2.56)	<.001*
Reflective motivation	3.70 (1.10)	1.40 (1.56)	2.91 (2.17–3.90)	<.001*
Automatic motivation	2.58 (1.51)	.60 (1.56)	2.28 (1.76–2.96)	<.001*

* *p* < .05.

### Multiple logistic regression analysis predicting screening attendance

A multiple logistic regression analysis was conducted in which all of the COM‐B model components were entered, along with the covariates of relationship status, employment status, and long‐standing illness status. Together, these variables significantly predicted screening attendance versus non‐attendance, *χ* ^2^(8) = 85.81, *p* < .001. Reflective motivation was the only COM‐B model component that was a significant independent predictor of screening attendance, such that increased reflective motivation scores increased the odds of having attended CC screening (OR, 2.37; 95% CI, 1.47–3.83). Having a long‐standing illness was also a predictor of not attending CC screening in this analysis (see Table 3).

**TABLE 3:** Summary of multiple logistic regression analysis predicting screening attendance.

Variable	Adjusted OR (95% CI)	*p*‐value
Relationship status	1.86 (.71–4.87)	.208
Employment status	.64 (.16–2.59)	.640
Long‐standing illness	.27 (.75–.99)	.049*
Psychological capacity	1.21 (.71–2.05)	.487
Social opportunity	.92 (.71–2.05)	.600
Physical opportunity	.83 (1.05–.67)	.829
Reflective motivation	2.37 (1.47–3.83)	<.001*
Automatic motivation	1.33 (.91–1.96)	.141

* *p* < .05.

### Sensitivity analysis

In total, 200 participants were included in the sensitivity analysis. This included 179 participants who had received a screening invitation and 21 who reported not receiving a screening invitation (see Table 4). We excluded the four participants who reported they were not sure, or did not know if they had received a screening invitation. Chi‐square and t‐test analyses were carried out to assess any significant differences between descriptive demographics of those not invited for CC screening (*n* = 21) and those invited for CC screening (*n* = 179). Those not invited were less likely to have attended screening (19% vs. 53.6%), be of white ethnicity (57.1% vs. 79.9%), or have had the HPV vaccination (61.8% vs. 70.9%), and more likely to be religious (52.4% vs. 27.9%) or have an existing illness (28.6% vs. 16.2%).

**TABLE 4:** Demographic descriptive data for sensitive analysis groups.

Variable	Full sample (*n* = 200)	Non‐invited participants only (*n* = 21)	Invited participants (*n* = 179)
	*n* (%)	*n* (%)	*n* (%)
Screening attendance*			
Yes	100 (50)	4 (19)	96 (53.6)
No	100 (50)	17 (81)	83 (46.4)
Gender			
Female	192 (96)	20 (95.2)	172 (96.1)
Prefer self‐identify	8 (4)	1 (4.8)	7 (3.9)
Ethnicity*			
White British or White Other	155 (77.5)	12 (57.1)	143 (79.9)
Other	45 (22.5)	9 (42.9)	36 (20.1)
Place of residence			
England	178 (89)	18 (85.7)	160 (89.4)
Scotland, Northern Ireland and Wales	22 (11)	3 (14.3)	19 (10.6)
Education			
Higher education	158 (79)	17 (81)	141 (78.8)
School‐level education	42 (21)	4 (19)	38 (21.2)
Relationship status			
Partnered	116 (58)	13 (61.9)	103 (57.5)
Other	84 (42)	8 (38.1)	76 (42.5)
Religion*			
Not religious	132 (66)	8 (38.1)	124 (69.3)
Religious	61 (30.5)	11 (52.4)	50 (27.9)
Prefer not to say^a^	7 (3.5)	2 (9.5)	5 (2.8)
Employment status			
Employed	137 (68.5)	16 (76.2)	121 (67.6)
Not employed	29 (14.5)	2 (9.5)	27 (15.1)
Missing^a^	34 (17)	3 (14.3)	31 (17.3)
Long‐standing illness, disability or infirmity*			
No	165 (82.5)	15 (71.4)	150 (83.8)
Yes	35 (17.5)	6 (28.6)	29 (16.2)
HPV vaccinated*			
Yes	140 (70)	13 (61.9)	127 (70.9)
No	49 (24.5)	8 (38.1)	41 (22.9)
Not sure	11 (5.5)	0 (0)	11 (6.1)
Number of doses*			
Two or more	106 (53)	9 (42.9)	97 (54.2)
One	9 (4.5)	2 (9.5)	7 (3.9)
Not sure^a^	85 (42.5)	10 (47.6)	23 (12.8)

	Mean (SD)	Mean (SD)	Mean (SD)
Age	27.60 (1.76)	27.19 (1.75)	27.65 (1.76)
Health status	2.51 (.78)	2.43 (.93)	2.5 (.77)

^a^ Don't know/not sure/missing responses were excluded from the analysis. Do not know/not sure responses regarding invitation status were also excluded.

* *p* < .05.

When including participants who had not received a CC screening invitation, age (OR, 1.21; 95% CI, 1.03–1.43), religious status (OR, .52; 95% CI, .28–.97) and HPV vaccination status (OR, 2.17; 95% CI, 1.11–4.23) now became significantly associated with screening attendance (see Supporting Information).

There were no changes regarding the COM‐B findings; all COM‐B components were also found to have a significant unadjusted association with CC screening attendance, while in the adjusted analysis, only reflective motivation remained statistically significant (see Supporting Information).

## DISCUSSION

CC screening uptake in the UK has declined in recent years, but remains the most effective way for early detection and treatment of CC (Urwin et al., 2024). The present study aimed to examine factors associated with screening attendance in young women and people with a cervix in the UK. Several demographic factors were identified as significantly associated with screening behaviour. The strongest demographic predictors for having attended screening were being employed or being partnered. In contrast, having a long‐standing illness or disability decreased the likelihood of utilising CC screening. Furthermore, in the sensitivity analysis only, those who were HPV vaccinated, older and not religious were more likely to have attended CC screening. This difference could be due to a smaller sample size in the main analysis and a more even distribution of attendance status; or it could reflect that the demographics and experiences that predict CC screening attendance are different for those who have not been invited for screening. For example, in our sample, those who reported not receiving an invitation were less likely to be of white ethnicity or HPV vaccinated, and more likely to be religious and have an existing illness, compared to the sample that reported receiving an invitation.

The findings regarding HPV vaccination show that it is possible that HPV vaccination may improve CC screening uptake by raising awareness of the link between HPV and CC. Being HPV vaccinated may also reinforce positive health behaviours and empower young women and people with a cervix to take up health screening invitations, as well as help reduce anxieties or offer reassurance about the likelihood of receiving positive screening results, thereby increasing attendance. On the contrary, this also highlights the possible negative impact of not receiving the HPV vaccination on CC screening attendance. Individuals may refuse HPV vaccination for a multitude of reasons (e.g. vaccine hesitancy and parental decision‐making) (Cherven et al., 2023; Grandahl et al., 2014), which may then also negatively impact future CC screening attendance.

Demographic factors associated with screening behaviour can also inform the development of interventions to increase the uptake of CC screening, for example, through tailoring interventions to target specific groups (e.g. ethnic minorities, single women etc.).

Previous research has identified ethnic minority groups as less likely to attend screening in the UK when compared to white British women (Marlow et al., 2015), with migration to the UK (not born in the UK) associated with disengagement with screening services. This is supported by the findings in our study that those who had not received a CC screening invitation had attributes consistent with ‘'harder‐to‐reach group'; for example, they were more likely to be from an ethnic minority, religious, have an existing illness, and less likely to be HPV vaccinated. This highlights possible unique barriers and associations to screening attendance in this group, who are missing out on key information regarding screening. Previous qualitative findings suggested that education from community members could be one way to improve awareness and uptake among these harder‐to‐reach groups (Brackertz, 2007; Shpendi et al., 2024).

It is also important to note that receiving a screening invitation was significantly associated with screening attendance in the sensitivity analysis. There can be a number of reasons why individuals may not receive a screening invitation letter. For example, errors made by a private sector company, Capita, contracted to provide GP support services, resulted in over 43,000 women not receiving an invitation letter (Torjesen, 2019). In addition, young people may move home frequently and therefore may miss invitation letters sent to a previous address. Although all women and people with a cervix are eligible by age and can still book and attend CC screening under the NHS, this may result in unique barriers to attendance in this subgroup. Nonetheless, motivations to attend may still be important in this subgroup, given that a number of participants in the current study who had not received an invitation letter reported having attended CC screening.

All of the COM‐B model components assessed in the current study were found to be associated with screening attendance. Thus, increased scores in psychological capability, social opportunity, physical opportunity, reflective motivation, and automatic motivation were associated with an increased likelihood of having attended screening. When all of the COM‐B model components and the demographic covariates were considered together, reflective motivation was the only COM‐B model component that was a significant predictor of screening behaviour. No difference was found in the sensitivity analysis, indicating consistency in the observed relationships between the COM‐B components and screening behaviour. Reflective motivation refers to the conscious, deliberate processes influencing behaviour, such as beliefs, intentions, values, and goals (Michie et al., 2011). The importance of intention in cancer screening has been reported in previous studies on colorectal cancer (Christou & Thompson, 2012), CC (Lahole et al., 2024; Ogilvie et al., 2013) and general cancers (Ewing et al., 2023), and is a prominent factor in many models of health behaviour, including the theory of planned behaviour (Ajzen, 1991) and protection motivation theory (Prentice‐Dunn & Rogers, 1986).

The current findings are consistent with previous literature (Bowyer et al., 2014; Shpendi et al., 2025) and highlight that reflective motivation factors could be used as targets for future interventions to improve screening uptake, such as increasing awareness of the benefits of screening or self‐efficacy. Understanding and targeting motivational components could be fundamental for improving screening uptake. A systematic review and meta‐analysis found that different motivational interventions could effectively improve CC screening behaviours (Pourebrahim‐Alamdari et al., 2020). However, a recent study found no evidence of its effectiveness. This may be something to do with how the intervention is delivered, with mailed interventions proving not as effective (Wilding et al., 2023). Furthermore, a meta‐analysis by Sheeran et al. (2016) found that interventions that successfully changed attitudes, norms, and self‐efficacy produced significant changes in intentions, suggesting that interventions targeting these factors might increase reflective motivation factors and attendance at CC screening. Webb and Sheeran (2008) also reported that interventions that successfully changed intentions produced significant changes in behaviour. Previous intervention studies have utilised motivational messages in improving cancer screening uptake (Chan & So, 2021). These findings showcase the potential for targeting motivational factors to increase the intention to participate and actual screening behaviours.

The Human Behaviour‐Change Project (HBCP) has sought to connect behaviour change techniques (BCTs) to underlying mechanisms of action (MoAs), helping identify specific BCTs that can effectively increase motivation (Carey et al., 2019; Connell et al., 2019). Understanding and targeting reflective motivation is crucial for developing interventions to increase CC screening uptake in young women and people with a cervix. For example, BCTs such as pros and cons, aiding mental rehearsals of the procedure, and emphasising self‐incentives through educational materials could be used to target reflective motivation to attend CC screening. Additional analyses in the current study (see Supporting Information) also highlighted which individual reflective motivation items were significantly associated with CC screening attendance. This additional analysis highlighted items referring to the “importance” of screening and screening being “worthwhile” as having significant associations with CC screening attendance. Future interventions could target these reflective motivational aspects in order to encourage CC screening attendance.

### Study strengths and limitations

The current study has several strengths. First, the questionnaire was based on previous qualitative work (Shpendi et al., 2024), and the COM‐B model provided a strong theoretical framework. Significant associations were found between the COM‐B components and CC screening attendance, with reflective motivation emerging as the key predictor of attendance behaviour. Second, the use of the online platform “Prolific” ensured a wide reach of participants for recruitment across the UK. Pre‐screening filters also allowed for accurate recruitment of the desired population and the identification of attendee and non‐attendee groups.

However, the study also had some limitations. First, the current study did not assess physical capability and therefore did not provide a full test of the COM‐B model. This was because previous qualitative research (Shpendi et al., 2024) with a similar population of interest did not identify physical capability as an important factor for CC screening attendance in young women in the UK. However, it is important to note that this study found that having a long‐standing illness or disability did significantly impact CC screening attendance. Although this study focused on previously highlighted COM‐B components, allowing for a timelier questionnaire and avoidance of participation burden (Aiyegbusi et al., 2022), future research could look to explore the impact of physical capability barriers in CC screening attendance.

Second, the sample comprised a predominantly White and educated majority, which may limit the generalisability of the results. For example, those from more marginalised groups may report more barriers related to social and physical opportunity than reported in the current study. Furthermore, the current study was powered to analyse the factors associated with screening behaviour in young women and people with a cervix and was therefore not powered to identify factors associated with CC screening attendance in individual subgroups (e.g., ethnic minority groups and lower socioeconomic status groups). For example, socioeconomic status has been associated with screening attendance for cervical (Wearn & Shepherd, 2022) and other (Schootman et al., 2006; Wells & Horm, 1998) cancers. However, in line with many models of health behaviour (Connor & Norman, 2015), the impact of socioeconomic status on screening attendance is likely to be mediated by social cognitive variables such as self‐efficacy and perceived psychological costs (Orbell et al., 2017). Nonetheless, future research should be adequately powered to allow subgroup analysis in order to establish whether the factors associated with CC screening behaviour are consistent or vary across different population subgroups.

Third, the use of convenience sampling can result in a rapid‐responder bias (Prolific, 2024); therefore, consideration was given to the time and day of the week that the study was launched. The study was made public outside of working hours (9 am–5 pm) to allow those with daytime commitments an opportunity to respond. Fourth, screening attendance and HPV vaccination were self‐reported. Given that accessing NHS records was not possible at this time to cross‐reference these reports and the nature of CC screening, it would be assumed that participants would remember having attended or not attended. However, due to some participants reporting ‘not sure’ regarding HPV vaccination doses, comparisons to population rates of vaccination could not be made.

## CONCLUSION

Reflective motivation factors are key in promoting CC screening attendance in young women. Future work should utilise BCTs, such as pros and cons and self‐incentives, in educational materials to increase reflective motivation to attend CC screening in young people. Demographic factors that can also significantly impact screening uptake should be considered when tailoring interventions to this age group. Addressing these factors may have a positive impact on boosting CC screening attendance among young women, consequently reducing future mortality rates.

## AUTHOR CONTRIBUTIONS

**Sonia Shpendi:** Conceptualization; methodology; formal analysis; data curation; funding acquisition; writing – original draft; writing – review and editing. **Paul Norman:** Conceptualization; methodology; formal analysis; writing – review and editing; supervision. **Jilly Gibson‐Miller:** Conceptualization; methodology; formal analysis; writing – review and editing; supervision. **Rebecca Webster:** Conceptualization; methodology; writing – review and editing; supervision.

## Supporting information


Data S1.

## Data Availability

The data that support the findings of this study are available from the corresponding author upon reasonable request.
