# Supplementary material for: Cervical screening attendance in young women and people with a cervix: An application of the COM‐B model
Source: Br J Health Psychol. 2025 Aug 18;30(3):e70016. doi: 10.1111/bjhp.70016 (PMC12361587; doi:10.1111/bjhp.70016)
Supplement: Supplementary file 1 [file BJHP-30-0-s001.odt]

Supplementary Table 1.  Full demographic frequencies

| Characteristics |  | Attendees n (%) | Non-attendees n (%) | Total n (%) |
| --- | --- | --- | --- | --- |
| Age |  |  |  |  |
|  | 25 | 12 (12.5) | 17 (20.5) | 29 (16.2) |
|  | 26 | 14 (14.6) | 12 (14.5) | 26 (14.5) |
|  | 27 | 17 (17.7) | 11 (13.3) | 28 (15.6) |
|  | 28 | 12 (12.5) | 17 (20.5) | 29 (16.2) |
|  | 29 | 16 (16.7) | 14 (16.9) | 30 (16.8) |
|  | 30 | 25 (26) | 12 (14.5) | 37 (20.7) |
| Gender |  |  |  |  |
|  | Female | 94 (97.9) | 78 (94) | 172 (96.1) |
|  | Trans Male/Trans Man | 0 (0) | 2 (2.4) | 2 (1.1) |
|  | Other Non-binary No gender Trans Masc | 2 (2.1) 1 (1.05) 1 (1.05) 0 | 3 (3.6) 2 (2.4) 1 (1.2) 0 | 5(2.8) |
| Ethnicity |  |  |  |  |
|  | White British or White Other | 80 (83.3) | 63 (75.9) | 143 (79.9) |
|  | Asian or Asian British | 10 (10.4) | 15 (18.1) | 25 (14) |
|  | Black, Black British, Caribbean or African | 2 (2.1) | 1 (1.2) | 3 (1.7) |
|  | Mixed or multiple ethnic groups | 4 (4.2) | 3 (3.6) | 7 (3.9) |
|  | Other ethnic groups (Arab and/or any other ethnic group) | 0 (0) | 1 (1.2) | 1 (0.6) |
| Place of residence |  |  |  |  |
|  | London | 15 (15.6) | 15 (18.1) | 30 (16.8) |
|  | Scotland | 3 (3.1) | 7 (8.4) | 10 (5.6) |
|  | Northern Ireland | 3 (3.1) | 2 (2.4) | 5 (2.8) |
|  | Wales | 2 (2.1) | 2 (2.4) | 4 (2.2) |
|  | Yorkshire and The Humber | 13 (13.5) | 8 (9.6) | 21 (11.7) |
|  | East of England | 12 (12.5) | 7 (8.4) | 19 (10.6) |
|  | North East (England) | 3 (3.1) | 2 (2.4) | 5 (2.8) |
|  | North West (England) | 13 (13.5) | 7 (8.4) | 20 (11.2) |
|  | West Midlands (England) | 5 (5.2) | 7 (8.4) | 12 (6.7) |
|  | East Midlands (England) | 7 (7.3) | 8 (9.6) | 15 (8.4) |
|  | South East (England) | 14 (14.6) | 12 (14.5) | 26 (14.5) |
|  | South West (England) | 6 (6.3) | 6 (7.2) | 12 (6.7) |
| Education |  |  |  |  |
|  | Undergraduate degree | 42 (43.8) | 27 (32.5) | 69 (38.5) |
|  | Postgraduate degree | 34 (35.4) | 27 (32.5) | 61 (34.1) |
|  | Higher certificates | 5 (5.2) | 4 (4.8) | 9 (5) |
|  | A-level, National Diploma or equivalent | 12 (12.5) | 15 (18.1) | 27 (15.1) |
|  | GCSE | 3 (3.1) | 8 (9.6) | 11 (6.1) |
|  | PhD | 0 (0) | 2 (2.4) | 2 (1.1) |
| Relationship status |  |  |  |  |
|  | Partnered | 45 (46.9) | 33 (39.8) | 78 (43.6) |
|  | Single | 30 (31.3) | 43 (51.8) | 73 (40.8) |
|  | Married | 18 (18.8) | 7 (8.4) | 25 (14) |
|  | Other | 3 (3.1) | 0 (0) | 3 (1.7) |
| Religion |  |  |  |  |
|  | Not religious | 70 (72.9) | 54 (65.1) | 124 (69.3) |
|  | Catholicism/Christianity | 19 (19.8) | 14 (16.9) | 33 (18.4) |
|  | Islam | 3 (3.1) | 10 (12) | 13 (7.3) |
|  | Prefer not say | 2 (2.1) | 3 (3.6) | 5 (2.8) |
|  | Buddhism | 0 (0) | 1 (1.2) | 1 (0.6) |
|  | Hinduism | 0 (0) | 0 (0) | 0 (0) |
|  | Judaism | 1 (1) | 1 (1.2) | 2 (1.1) |
|  | Paganism | 1 (1) | 0 (0) | 1 (0.6) |
| Employment status |  |  |  |  |
|  | Full-Time | 65 (67.7) | 39 (47) | 104 (58.1) |
|  | Part-Time | 9 (9.4) | 8 (9.6) | 17 (9.5) |
|  | Unemployed (and job seeking) | 4 (4.2) | 11 (13.3) | 15 (8.4) |
|  | Not in paid work (e.g. homemaker, retired or disabled) | 5 (5.2) | 7 (8.4) | 12 (6.7) |
|  | Due to start within the next month | 0 (0) | 0 (0) | 0 (0) |
|  | Missing data/ data expired | 13 (13.5) | 18 (21.7) | 31 (17.3) |
| Health status |  |  |  |  |
|  | Good | 60 (62.5) | 46 (55.4) | 106 (59.2) |
|  | Fair | 22 (22.9) | 21 (25.3) | 43 (24) |
|  | Excellent | 14 (14.6) | 10 (12) | 24 (13.4) |
|  | Poor | 0 (0) | 6 (7.2) | 6 (3.4) |
| Longstanding illness, disability or infirmity that limits daily activities |  |  |  |  |
|  | No | 87 (90.6) | 63 (75.9) | 150 (83.8) |
|  | Yes | 9 (9.4) | 20 (24.1) | 29 (16.2) |

Supplementary Table 2.  Associations between individual reflective motivation items and screening attendance 

| Reflective Motivation Items | Attendees | Non-attendees | Total | Unadjusted OR (95% CI) |
| --- | --- | --- | --- | --- |
|  | Mean  (SD) | Mean  (SD) | Mean  (SD) |  |
| I am motivated to attend cervical cancer screening | 3.22 (2.55) | -1.08 (3.22) | 1.07 (3.61) | 1.54 (1.37 - 1.73)* |
| Cervical cancer screening is important | 4.72 (0.74) | 3.19 (2.14) | 3.96 (1.77) | 2.26 (1.66 - 3.08)* |
| I do not perceive myself to be at risk of cervical cancer | 2.59 (1.94) | 2.48 (1.82) | 2.54 (1.88) | 1.03 (0.89 - 1.20) |
| I am no more at risk of cervical cancer than anyone my age | 2.49 (2.02) | 2.35 (1.98) | 2.42 (2.00) | 1.04 (0.90 - 1.91) |
| I intend to go to my cervical cancer screening | 4.17 (1.82) | -0.21 (3.55) | 1.98 (3.57) | 1.71 (1.47 - 1.99)* |
| I intend to go to my cervical cancer screening every time I am invited | 3.92 (2.08) | -0.91 (3.46) | 1.51 (3.74) | 1.66 (1.45 - 1.90)* |
| Cervical cancer screening is not a current priority for me | 3.37 (1.76) | 2.83 (1.67) | 3.10 (1.74) | 1.20 (1.02 - 1.42)* |
| I believe attending my cervical cancer screening is worthwhile | 4.43 (1.23) | 1.81 (3.18) | 3.12 (2.74) | 1.81 (1.47 - 2.22)* |
| I believe attending my cervical cancer screening will benefit me | 4.18 (1.42) | 1.60 (3.26) | 2.89 (2.82) | 1.61 (1.36 - 1.91)* |
| I have had previous negative experiences with health-related screening or at the GP | 3.65 (1.62) | 3.23 (1.71) | 3.44 (1.67) | 1.17  (0.98 – 1.38) |

Note. * p < .05. 

Supplementary Table 3. Demographic data and associations with screening attendance (including those who have not received a screening invitation)

| Variable |  | Attendees | Non- attendees | Total | Unadjusted OR        (CI 95%) | p |
| --- | --- | --- | --- | --- | --- | --- |
|  |  | n | n | n (%) |  |  |
| Gender | Female | 98 | 94 | 192 (96) | 3.13 (0.62 - 15.89) | .17 |
|  | Prefer self-identify | 2 | 6 | 8 (4) |  |  |
| Ethnicity | White British or White  Other | 84 | 71 | 155 (77.5) | 2.14 (1.08 - 4.26) | .03* |
|  | Other | 16 | 29 | 45 (22.5) |  |  |
| Place of residence | England | 90 | 88 | 178 (89) | 1.23 (0.50 - 3.00) | .65 |
|  | Scotland, Northern Ireland and Wales | 10 | 12 | 22 (11) |  |  |
| Education | Higher education | 84 | 74 | 158 (79) | 1.85 (0.92- 3.70) | .85 |
|  | School level education | 16 | 26 | 42 (21) |  |  |
| Relationship status | Partnered | 65 | 51 | 116 (58) | 1.78 (1.01 - 3.15) | .046* |
| Other | 35 | 49 | 84 (42) |  |  |
| Religion | Not Religious | 73 | 59 | 132 (66) | 0.52 (0.28 - 0.97) | .04* |
|  | Religious | 24 | 37 | 61 (30.5) |  |  |
|  | Prefer not to say a | 3 | 4 | 7 (3.5) |  |  |
| Employment status | Employed | 76 | 61 | 137 (68.5) | 2.77 (1.18 - 6.52) | .02* |
| Not Employed | 9 | 20 | 29 (14.5) |  |  |
|  | Missing a | 15 | 19 | 34 (17) |  |  |
| Longstanding illness, disability or infirmity | No | 90 | 75 | 165 (82.5) | 0.33 (0.15 - 0.74) | .007* |
| Yes | 10 | 25 | 35 (17.5) |  |  |
| Received screening invitation letter | Yes | 96 | 83 | 179 (89.5) | 5.40 (1.50 - 19.43) | .01* |
| No | 3 | 14 | 17 (8.5) |  |  |
| Don’t know / Not sure a | 1 | 3 | 4 (2) |  |  |
| HPV vaccinated | Yes | 78 | 62 | 140 (70) | 2.17 (1.11 - 4.23) | .02* |
| No | 18 | 31 | 49 (24.5) |  |  |
| Not sure | 4 | 7 | 11 (5.5) |  |  |
| Number of doses | Two or more | 62 | 44 | 106 (53) | 2.82 (0.67 - 11.88) | .158 |
| One | 3 | 6 | 9 (4.5) |  |  |
|  | Not sure a | 13 | 12 | 25 (12.5) |  |  |
|  |  | Mean (SD) | Mean (SD) | Mean (SD) |  |  |
| Age |  | 27.89 (1.75) | 27.31 (1.73) | 27.60 (1.76) | 1.21 (1.03-1.43) | .02* |
| Health status |  | 2.47 (0.75) | 2.56 (0.82) | 2.52 (0.78) | 0.86 (0.60 - 1.23) | .42 |
| Note. * p < .05. a Don’t know/Not sure/Missing responses were excluded from the analysis. |

Supplementary Table 4. COM-B components and associations with screening attendance (including those who have not received a screening invitation)

| Variable | Attendees | Non-attendees | Unadjusted OR (95% CI) | P - value |
| --- | --- | --- | --- | --- |
|  | Mean (SD) | Mean (SD) |  |  |
| Psychological Capacity | 3.31 (1.00) | 2.42 (1.37) | 1.90 (1.45 - 2.50) | <.001* |
| Social Opportunity | 2.45 (1.73) | 0.95 (2.15) | 1.48 (1.26 - 1.74) | <.001* |
| Physical Opportunity | 3.84 (1.18) | 2.69 (1.33) | 1.98 (1.56 - 2.52) | <.001* |
| Reflective Motivation | 3.67 (1.12) | 1.53 (1.62) | 2.71 (2.07 - 3.54) | <.001* |
| Automatic Motivation | 2.55 (1.50) | 0.70 (1.74) | 2.01 (1.61 - 2.51) | <.001* |
| Note. * p < .05. |  |

Supplementary Table 5. Summary of multiple logistic regression analysis predicting screening attendance (including those who have not received a screening invitation). 

| Variable | Adjusted OR (95% CI) | p - value |
| --- | --- | --- |
| Age | 1.57 (1.10 - 2.22) | .011* |
| Ethnicity | 0.71 (0.17 - 3.00) | .639 |
| Relationship Status | 3.30 (1.03 - 10.34) | .044* |
| Religion | 0.54 (0.17 -1.77) | .308 |
| Employment | 0.82 (0.16 - 4.10) | .809 |
| Longstanding Illness | 0.39 (0.10 - 1.67) | .204 |
| Screening Invitation | 26.57 (2.10 - 340.15) | .012* |
| HPV Vaccination | 3.10 (0.83 - 11.64) | .094 |
| Psychological Capacity | 1.18 (0.63 - 2.20) | .610 |
| Social Opportunity | 0.82 (0.56 - 1.22) | .331 |
| Physical Opportunity | 1.42 (0.83 - 2.41) | .198 |
| Reflective Motivation | 2.79 (1.65 - 4.72)* | <.001* |
| Automatic Motivation | 1.16 (0.75 - 1.80) | .506 |
| Note. * p < .05. |  |

Supplementary Table 6.  Associations between individual reflective motivation items and screening attendance (including those who have not received a screening invitation). 

| Reflective Motivation Items | Attendees | Non-attendees | Total | Unadjusted OR (95% CI) | p-value |  |
| --- | --- | --- | --- | --- | --- | --- |
|  | Mean  (SD) | Mean  (SD) | Mean  (SD) |  |  |  |
| I am motivated to attend cervical cancer screening | 3.28 (2.53) | -1.29 (3.15) | 1.16 (3.64) | 1.58 (1.40 - 1.79) | <.001* |  |
| Cervical cancer screening is important | 4.47 (0.73) | 3.13 (2.14) | 3.99 (1.74) | 2.43 (1.72 - 3.41) | <.001* |  |
| I do not perceive myself to be at risk of cervical cancer | 2.57 (1.93) | 2.55 (1.77) | 2.56 (1.85) | 1.01 (0.86 - 1.18) | 0.946 |  |
| I am no more at risk of cervical cancer than anyone my age | 2.52 (2.02) | 2.37 (2.03) | 2.45 (2.02) | 1.04 (0.90 - 1.20) | 0.625 |  |
| I intend to go to my cervical cancer screening | 4.22 (1.78) | -0.55 (3.55) | 2.01 (3.64) | 1.75 (1.49 – 2.06) | <.001* |  |
| I intend to go to my cervical cancer screening every time I am invited | 3.96 (2.09) | -1.40 (3.31) | 1.47 (3.81) | 1.73 (1.50 - 2.00) | <.001* |
| Cervical cancer screening is not a current priority for me | 3.39 (1.74) | 2.98 (1.68) | 3.20 (1.72) | 1.15 (0.97 - 1.37) | 0.113 |  |
| I believe attending my cervical cancer screening is worthwhile | 4.46 (1.42) | 1.63 (3.19) | 3.15 (2.74) | 1.93 (1.53 - 2.42) | <.001* |  |
| I believe attending my cervical cancer screening will benefit me | 4.19 (1.42) | 1.40 (3.28) | 2.89 (2.74) | 1.66 (1.39 - 1.99) | <.001* |  |
| I have had previous negative experiences with health-related screening or at the GP | 3.68 (1.61) | 3.23 (1.71) | 3.47 (1.67) | 1.18  (0.98 – 1.41) | 0.074 |  |

Supplementary Material A. Main questionnaire

Demographic section:

- What is your Prolific ID?

Please note that this response should auto-fill with the correct ID: 

- Please specify your ethnicity - select one category that you feel best represents your ethnicity

Asian or Asian British (Indian, Pakistani, Bangladeshi, Chinese and/or Any other Asian background)

Black, Black British, Caribbean or African (Caribbean, African and/or Any other Black, Black British, or Caribbean background)

Mixed or multiple ethnic groups (White and Black Caribbean, White and Black African, White and Asian and/or Any other Mixed or multiple ethnic background) 

White (English, Welsh, Scottish, Northern Irish or British, Irish, Gypsy or Irish Traveller, Roma and/or Any other white background)

Other ethnic group (Arab and/or any other ethnic group)

Please specify further:_________

- What gender do you identify as? 

Male

Female 

Trans/Male/Trans Man

Trans Female/Trans Women

Other (please answer in the space given)

Prefer not to answer

- What is the highest degree or level of education you have completed?

GCSE 

A-level, National Diploma or equivalent

Undergraduate degree

Higher certificates (including Advanced Apprenticeship/Diplomas)

Postgraduate degree (including all master’s degrees)

PhD 

Other:_______

- What is your current relationship status?

Single 

Married 

Partnered 

Other

- What religion do you identify most with?

Catholicism/Christianity

Judaism

Islam

Buddhism

Hinduism 

Prefer not to say 

Not religious

Other:______

- How would you rate your health status?

Poor

Fair

Good

Excellent 

- Do you have a longstanding illness, disability or infirmity that limits daily activities in any way, compared to people of the same age? 

Yes

No

- Have you had the HPV vaccine? (This is a vaccine to protect against viruses that can cause cervical cancer. It is usually given in two doses (aged 12/13 or school year 8) or free for anyone under 25 years old)

Yes

No

Not sure

	7a. If Yes, How many doses of the vaccine have you received?

One dose 

Two or more doses

Don’t know/not sure

COM-B items section

Scale: Strongly disagree - Strongly agree (rate -5 - 5 (10 response options)

Capability

 I am psychologically able to attend cervical cancer screening. 

(What is psychological capability? Knowledge and/or psychological skills, strength or stamina to engage in the necessary thought processes to attend cervical cancer screening)

- I know what cervical cancer screening is for

- I know why cervical cancer screening is needed

- I know how cervical cancer screening is carried out

- Cervical cancer screening is something you do when you are older (35+)

- Cervical cancer screening is not needed at my age

- I know about the link between the HPV vaccination and cervical cancer

- I take into consideration my HPV vaccination status when deciding whether or not to attend my cervical cancer screening

- I know how to book a cervical cancer screening appointment

- I know where to find information about cervical cancer screening

Opportunity

I have the social opportunity to attend a cervical cancer screening. 

(What is social opportunity? Interpersonal influences, social cues and cultural norms provide the opportunity to attend cervical cancer screening, e.g support from friends and family)

- I am aware of others in my social circle/community who have attended their cervical cancer screening

- I am comfortable discussing cervical cancer screening experiences with my social circle

- In the past, I have learnt about cervical cancer screening from friends and family

- Someone in my family or close to me has been diagnosed with cancer

- I have received encouragement to attend my cervical cancer screening from my GP

- I value encouragement from my GP to attend my cervical cancer screening

I have the physical opportunity to attend cervical cancer screening. 

(What is the physical opportunity? The environment provides the opportunity to engage in cervical cancer screening, e.g. sufficient time, the necessary materials, reminders)

- Booking a cervical cancer screening is difficult for me

- It’s easy for me to book a cervical cancer screening appointment 

- I am too busy to attend a cervical cancer screening

- I am too busy to book a cervical cancer screening 

Motivation 

I am motivated to attend cervical cancer screening. 

(What is motivation? Conscious planning and evaluations (beliefs about what is good and bad, e.g. I have the desire to, I feel the need to) about cervical cancer screening)

- Cervical cancer screening is important

- I do not perceive myself to be at risk of cervical cancer 

- I am no more at risk of cervical cancer than anyone my age

- I intend to go to my cervical cancer screening

- I intend to go to my cervical cancer screening every time I am invited

- Cervical cancer screening is not a current priority for me

- I believe attending my cervical cancer screening is worthwhile

- I believe attending my cervical cancer screening will benefit me

- I have had previous negative experiences with health-related screening or at the GP

Attending cervical cancer screening is something that I do automatically.

(Automatic motivation involves doing something (or not) without giving it much thought or based on strong emotions e.g ‘is something I do before I realise I’m doing it”)

- Cervical cancer screening is embarrassing for me

- I am scared of the cervical cancer screening procedure

- Attending my cervical cancer screening would reassure me about my health

- I expect everything will be OK if attend my cervical cancer screening

- If I attend my cervical cancer screening I feel optimistic about my health in the future
